# Macrophages in Glioblastoma Development and Therapy: A Double-Edged Sword

**DOI:** 10.3390/life12081225

**Published:** 2022-08-12

**Authors:** Mengwan Wu, Ying Shi, Luyi Zhu, Luoyi Chen, Xinchen Zhao, Chuan Xu

**Affiliations:** 1 Integrative Cancer Center & Cancer Clinical Research Center, Sichuan Cancer Hospital & Institute, Sichuan Cancer Center, School of Medicine, University of Electronic Science and Technology of China, Chengdu 610041, China; 2 Department of Radiation Oncology, Sichuan Cancer Hospital, Chengdu 610041, China; 3 Key Laboratory of Cellular Physiology (Shanxi Medical University), Ministry of Education, Taiyuan 030001, China; 4 Department of Physiology, Shanxi Medical University, Taiyuan 030001, China

**Keywords:** glioblastoma, macrophage, microglia, immunotherapy, targeted therapy

## Abstract

Glioblastoma (GBM) is one of the leading lethal tumors, featuring aggressive malignancy and poor outcome to current standard temozolomide (TMZ) or radio-based therapy. Developing immunotherapies, especially immune checkpoint inhibitors, have improved patient outcomes in other solid tumors but remain fatigued in GBM patients. Emerging evidence has shown that GBM-associated macrophages (GAMs), comprising brain-resident microglia and bone marrow-derived macrophages, act critically in boosting tumor progression, altering drug resistance, and establishing an immunosuppressive environment. Based on its crucial role, evaluations of the safety and efficacy of GAM-targeted therapy are ongoing, with promising (pre)clinical evidence updated. In this review, we summarized updated literature related to GAM nature, the interplay between GAMs and GBM cells, and GAM-targeted therapeutic strategies.

## 1. Introduction

Glioblastoma (GBM) represents the most common primary intracranial tumor, accounting for approximately 48.6% of brain malignancies, according to the CBTRUS statistical report in 2020 [1]. GBM patients have a poor prognosis and a short survival time. Standard treatment for GBM consists of maximal safe surgical resection, followed by fractionated radiotherapy with concurrent or subsequent adjuvant temozolomide (TMZ)-based chemotherapy [2]. However, the response to current treatment remains limited in GBM patients.

In recent decades, immune-therapeutic approaches have achieved great clinical benefits in various solid tumors by restoring silenced or broken antitumor immune responses. However, favorable outcomes toward immunotherapy failed to be obtained in GBM patients, which is attributed to the unique intracranial environment of GBM. Compared to peripheral organs, the lack of a lymphatic network in the brain parenchyma and the existence of extensive vascular structures, including the blood–meningeal barrier (BMB), blood–cerebrospinal fluid barrier (BCSFB), and blood–brain barrier (BBB), endow GBM with “immune privilege” [3,4]. In this situation, GBM-associated macrophages (GAMs) are responsible for immunological surveillance and are comprised of ontogenetically distinct macrophage populations, including resident microglia and bone marrow-derived macrophages. During tumor evolution, GBM cells establish crosstalk with GAMs, stressing them into distinct phenotypes to affect tumor malignancy, vascular information, treatment response, and so on. In this review, updated research on the functional mechanism of GAMs is summarized, as well as related GAM-targeted therapy in GBM.

## 2. Biocharacters of GAMs

**Figure 1:**
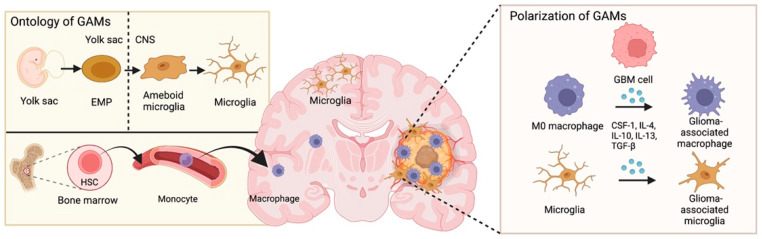
**Ontology and polarization of glioblastoma-associated macrophages.** Microglia originate from erythromyeloid progenitors (EMP) in the yolk sac. EMP-derived cells are incorporated into the CNS and take on an amoeboid morphology followed by transitioning toward a ramified state. GBM-associated macrophages are derived from monocytes, which arise from hematopoietic stem cells (HSCs) in bone marrow. In the GBM microenvironment, macrophages and microglia are induced by GBM-derived cytokines (such as CSF-1, IL-4/10/13, and TGF-β) and polarized into GAMs.

GAMs represent a mixed-cell collection exhibiting distinct ontology and phenotype, which can be provided by both intracranial microglia and macrophages from bone marrow (Figure 1). Microglia belong to the brain’s primary innate immune cells, originating from the primitive macrophage pool in the yolk sac. Microglia play a vital role in maintaining brain homeostasis by sensing environmental changes, removing cell debris, and providing neurotrophic factors [5].

Both the quantity and molecular characteristics of GAMs are highly plastic [6,7]. Evidence from single-cell sequencing demonstrated that GAMs constituted 59.05% and 27.87% of immunocytes in primary and recurrent GBMs, respectively [8]. The phenotype and activation state of GAMs are affected by multiple signaling molecules, growth factors, transcription factors, and epigenetic and posttranscriptional modifications [9,10,11]. Under tumoral or infective stimulation, unpolarized macrophages (M0 state) can be activated and polarized into two major subtypes, proinflammatory M1 and anti-inflammatory M2. M1 macrophages are characterized by increased secretion of proinflammatory cytokines, such as interleukin-1β (IL-1β), TNF, IL-12, and IL-18, which show strong antibacterial performance in mediating resistance to pathogens but can also lead to tissue destruction. Factors involved in proinflammation and immune stimulation are abundant in M1 GAMs, such as major histocompatibility complex class II (MHC-II), CD68 markers, CD80, and CD86 costimulatory molecules. In contrast, the M2 phenotype is generally supposed to participate in immunosuppression and tumor promotion, which is formed after being exposed to macrophage colony-stimulating factor 1 (CSF-1), interleukin 4 (IL-4), IL-10, and IL-13 [6,7]. For example, the expression of AEG-1 was positively associated with M2 markers in GBM tissues. Silencing AGE-1 in GBM decreased the M2 polarization of microglia and secretion of the tumor-supportive cytokines IL-6 and TGF-β1 [12]. However, accelerating findings have revealed that there are other versatile states of GAMs except for M1 or M2 phenotypes; a batch of GAMs performs as a mixture of M1 and M2 phenotypes [13]. M2 macrophages are divided into four subtypes: the M2a, M2b, M2c, and M2d subsets. The M2a subtype is activated by IL-4 and IL-13. M2b is elicited by IL-1R ligands or exposure to immune complexes plus LPS. M2c is induced by IL-10 and TGF-β [14], while M2d releases IL-10 and VEGF upon induction by TLR antagonists [15]. Among them, M2c GAMs are most closely related to GBM immune regulation, matrix deposition, and tissue remodeling [16]. Moreover, a nonpolarized M0 phenotype has also been observed in GAMs from GBM patients [14].

The abundance of intracranial GAMs predominantly determines the nature of the tumor immune environment. The M2-like GAM subtype plays a supportive role in constructing an immunosuppressive microenvironment by releasing inhibitory cytokines and chemokines to the antitumor immune response. When exposed to GBM-initiating cell-secreted factors, mTOR-STAT3-NF-κB signaling is activated and drives an immunosuppressive phenotype formation in microglia. Correspondingly, the infiltration and proliferation of effector T-cells are inhibited to block immune reactivity [17]. Broken secretion of CXCL9 and CXCL10 by GAMs suppresses T-cell infiltration into GBM tumors [18]. In addition, recruitment of M2 GAMs is accompanied by upregulated immune checkpoints, e.g., PD-L1, PD-L2, CD80, and CD86. An immune-exhausted state is formed, leading to an unsatisfactory response to anti-PD-1 therapy [18].

## 3. GAMs in Regulating Malignancy of GBM

Under normal conditions, microglia guarantee the intracranial steady state by sensing environmental changes, immune surveillance, and homeostatic maintenance [19,20]. These functions can be impaired by GBM cells to permit tumor initiation or growth, as verified by comparing the transcriptome of microglia from GBM-bearing mice and normal mice. A collection of genes encoding receptors that recognize various antigens, chemokines, and cytokines are downregulated in GAMs, corresponding to less sensitive microglia and impaired immune surveillance [21]. The homeostatic status of microglia can be disrupted by blocked SMAD3 signaling, leading to malfunction of self-renewal and grid-like distribution [22,23]. Increased PD-L1/PD-L2 expression has also been detected in microglia from GBM, suggesting enhanced immunologic tolerance in microglia [21].

**Figure 2:**
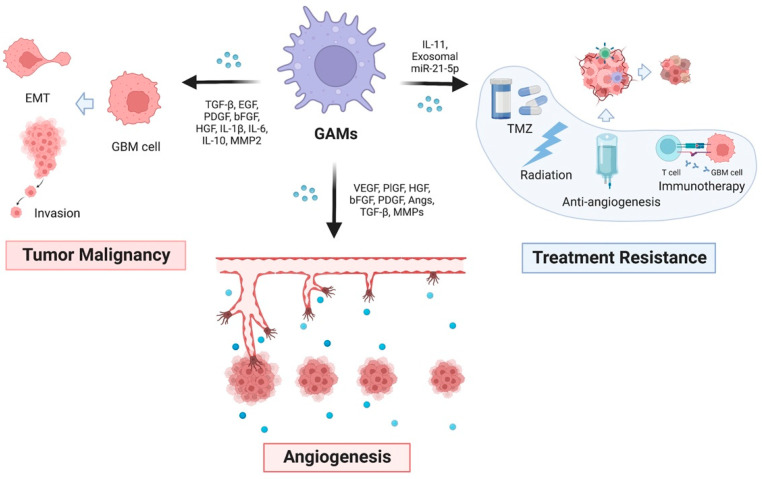
**Functions of glioblastoma-associated macrophages.** GAMs release many cytokines that promote the malignant phenotype of GBM, including tumor malignancy, angiogenesis, and treatment resistance (TMZ chemotherapy, radiation, anti-angiogenesis, and immunotherapy).

During tumor development, GAMs continuously exert their significant protumorigenic functions through various cytokines, including transforming growth factor-beta (TGF-β), epidermal growth factor (EGF), platelet-derived growth factor (PDGF), basic fibroblast growth factor (bFGF), hepatocyte growth factor (HGF), IL-1β, IL-6, IL-10, and matrix metallopeptidase-2 (MMP-2) (Figure 2). Among these, the effect of GAM-derived TGF-β on GBM has been extensively studied. Liu et al. found that TGF-β secreted by M2 phenotype GAMs upregulated phosphorylation of SMAD2/3, promoting epithelial-mesenchymal transition (EMT) and invasion of GBM cells [24]. In addition, GAMs participate in reciprocal molecular crosstalk with GBM stem cells (GSCs), displaying a more direct protumorigenic function by secreting TGF-β [25]. For example, integrin αvβ5 on GSCs can bind with TGF-β derived from GAMs in a paracrine way. Once combined, Src-STAT3 signaling is activated for protumorigenic effects.

GAM-derived TGF-β can also affect SMAD independently. For example, TGF-β signaling prevents proteasomal degradation of Sry-related high mobility group box (Sox) 9. Stabilization of Sox9 enhanced the migration and invasion of GBM cells, while downregulation of Sox9 inhibited the proliferation and development of xenograft GBM. After being activated by GM-CSF, GAMs can release the chemokine C-C ligand 5 (CCL5) to upregulate MMP2 secretion in GBM cells, consequently promoting tumor migration and invasion [26]. These findings may explain why the proliferation and migration of GBM cells were increased in the presence of microglia [27].

Infiltrating GAMs secrete IFNγ and elicit epigenetic immunoediting with stable expression of the myeloid-affiliated transcriptional program in GBM cells, which in turn leads to increased recruitment of GAMs. Moreover, similar epigenetic and transcriptional signatures have been identified in human mesenchymal subtype GSCs, which could indicate that epigenetic immunoediting may drive an acquired immune evasion program in the most aggressive mesenchymal GBM subtype by reshaping the tumor immune microenvironment [28].

## 4. GAMs in Angiogenesis of GBM

Microvascular hyperplasia is another hallmark of GBM, characterized by distorted vessels consisting of abnormal endothelial walls and mural cell (smooth muscle cells and pericytes) coverage. Normally, angiogenesis contains a series of steps, including breakdown of the basement membrane, remodeling of the extracellular matrix (ECM), and activation, proliferation, migration, and stabilization of endothelial cells. The best-characterized angiogenic factors include VEGFs, placental growth factor (PlGF), hepatocyte growth factor (HGF), basic fibroblast growth factor (bFGF), platelet-derived growth factor (PDGF), angiopoietins (Angs), TGF-β, and MMPs [29,30,31,32]. These neovessels formed even in hypoxic and necrotic areas of GBM and greatly support tumor cell growth and migration. However, highly proliferative GBM cells apart from vessels undergo extreme hypoxia and induce abnormal angiogenesis. These large and cross-linked pathological vessels are abnormal and functionally immature, resulting in exacerbated hypoxia with increased interstitial pressure (Figure 2).

There is a mutual effect between GAMs and angiogenesis. GAMs in hypoxic areas potently promote angiogenesis by secreting multiple angiogenic factors. These proangiogenic factors not only promote angiogenesis directly but also induce M2 polarization of GAMs, which contribute to further tumor angiogenesis. Hypoxia can induce the expression of hypoxia-inducible factor-1α (HIF-1α) in GAMs, a major proangiogenic factor that also significantly upregulates VEGF and VEGFR [33]. Interacting with VEGFR on endothelial cells stimulates MMP secretion to dissolve basement membrane and ECM components, which destabilizes endothelial-pericyte contact and facilitates the proliferation and migration of endothelial cells [34]. Beyond this, VEGF-α can foster the development of GAMs, interfere with the maturation of dendritic cells, limit T-cell recruitment into tumors, or promote T-cell exhaustion [35,36]. In particular, VEGF/VEGFR signaling induces TGF-β production in GAMs and promotes M2-like polarization [37]. In addition to VEGF, other proangiogenic factors are also implicated in tumor angiogenesis and immune suppression within the TME. Angiopoietin 2 (ANGPT2) negatively influences tumor immunity by stimulating Tie-2-expressing monocytes/macrophages to secrete IL-10, leading to the expansion of Treg cells and inhibiting effector T-cell activation [38,39]. Placental growth factor (PlGF), another member of the VEGF family, also promotes tumor-associated macrophage (TAM) repolarization to the M2 subtype. In a mouse glioma model, dual blockade of ANGPT2 and VEGF with the bispecific antibody A2 V has been shown to reprogram GAMs toward the antitumor M1 polarized subtype [40,41]. PlGF blockade induces vessel normalization and macrophage polarization from an M2-like to an M1-like phenotype [42,43]. Therefore, antiangiogenic therapy potentiates the normalization of the tumor immune microenvironment and may improve the effectiveness of immunotherapy during combination treatment [36]. Therapeutically, in the TAVAREC trial (NCT01164189), the monoclonal antibody bevacizumab targeting VEGF with TMZ chemotherapy has been shown to improve PFS in GBM compared to TMZ alone [44].

Conditionally, M2-like GAMs can mediate angiogenesis in a VEGF-independent manner. Cat Eye Syndrome Critical Region Protein 1 (CECR1) is highly expressed by M2-like macrophages in GBM and is positively correlated with tumoral microvascular density. The proangiogenic properties of CECR1 in macrophages were partially mediated via paracrine activation of pericytes by PDGFB-PDGFRβ signaling. CECR1-PDGFB-PDGFRβ cross-activation between macrophages and pericytes promoted pericyte recruitment and tumor angiogenesis [45]. In addition, GBM cell-derived IL-8 and CCL2 stimulated GAMs to secrete TNF-α and activated endothelial cells (ECs), characterized by the expression of VCAM-1, ICAM-1, CXCL5, and CXCL10. EC activation was associated with a higher WHO grade of GBM, worse overall survival (OS), and resistance to antiangiogenic therapy. Inhibition of TNFα prevented EC activation and prolonged survival of GBM-bearing mice [46]. Additionally, M2-polarized microglia released insulin-like growth factor-binding protein 1 (IGFBP1), which was induced by upregulation of macrophage colony-stimulating factor (MCSF) in a spleen tyrosine kinase (SYK)-PI3K-NFκB-dependent manner in GBM and promoted angiogenesis. Silencing IGFBP1 in microglial cells reduced the ability to induce angiogenesis, which might be a promising target for macrophage-based antiangiogenic therapy [47].

In addition to angiogenesis, at least four other modalities involved in neovascularization in GBM have been proposed: vascular co-option, vasculogenesis, vascular mimicry, and glioblastoma-endothelial cell transdifferentiation. Among these modalities, the role of GAMs in vasculogenesis has been investigated. Vasculogenesis involves the differentiation of circulating endothelial progenitor cells (EPCs). Apart from EPCs, GAMs express CXCR4 and migrate in response to the chemokine stromal cell-derived factor 1α (SDF-1α) gradient into tumor sites to contribute to vasculogenesis [48].

## 5. GAMs in Drug Resistance of GBM

Resistance to TMZ remains a main clinical challenge in most GBM patients [49]. Apart from the genetic nature of GBM cells, accumulating evidence has demonstrated that GAMs are closely related to the clinical response to TMZ by releasing various soluble factors [50,51]. Both microglia and macrophages are responsible for secreting IL-11, which in turn activates STAT3-MYC signaling in GBM, conferring TMZ resistance [50]. Inhibition of GAM recruitment and IL-11 secretion by ablation or genetic inactivation of myeloid-specific phosphoinositide-3-kinase gamma isoform (PI3Kγ) reversed TMZ sensitivity in a murine glioblastoma model [50]. Other researchers highlighted the potentially distinct effects of different GAM subpopulations in altering treatment responses. For example, M2-like GAMs contribute to resistance against TMZ by secreting exosomal miR-21-5p. Downregulating miR-21-enriched exosomes from M2 GAMs successfully overcame TMZ resistance in patient-derived xenograft (PDX) models [52]. Meanwhile, induction of M1-like polarization of GAMs by GBM-derived extracellular HMGB1 restored the sensitivity of GBM to TMZ [53]. Apart from chemoresistance, studies have suggested that GAMs are also involved in resistance to radiotherapy and antiangiogenic therapy. The dynamics and plasticity of the GAM transcriptome during radiotherapy correspond with an altered quantity of monocyte-derived macrophages or microglia [54]. Adding GAM inhibitors to standard treatment is supposed to recover the response. Crosstalk between GAMs and GSCs has been shown to be closely associated with GBM malignant behavior and therapeutic resistance. Pleiotrophin (PTN) has also been observed to be secreted by GAMs to stimulate GSCs through its receptor PTPRZ1, supporting GSC maintenance and tumorigenic potential to promote malignancy of GBM [55]. The influence of GAMs on GSCs is mediated via paracrine signaling by exosomes derived from GAMs. Small extracellular vesicles (sEVs) derived from GAMs transferred miR-27a-3p, miR-22-3p, and miR-221-3p to GSCs, triggering the pro-neural-to-mesenchymal transition in GSCs and increasing radiotherapy resistance [56] (Figure 2).

Since the famous Checkmate 143 trial (NCT02017717) demonstrated a restricted efficacy of nivolumab in improving the OS of GBM patients, great efforts have been made to elucidate the mechanism of immune checkpoint blockade (ICB) resistance [57]. The first explanation is that once PD-1 and CTLA-4 are blocked, GAMs are induced to express PD-L1 to interact with CD80, an alternative binding partner of PD-L1 on T-cells, thereby leading to CD4+ T-cell suppression, Treg expansion, and thus ICB resistance [58]. Therefore, the triple ICB regimen (supplementing anti-PD-L1) resulted in decreased tumor growth and an enhanced response compared with the double ICB regimen (11/13 vs. 6/13) [58]. Studies conducted by Goswami S et al. demonstrated that a unique population of CD73 high-expressing macrophages persisted after ICB treatment. Knocking out CD73 prolonged survival in a murine model of GBM treated with anti-PD-1 and anti-CTLA-4, which provided potential rationales for combining macrophage-targeted therapy with ICB [59]. The PI3K/Akt pathway is critical in modifying the polarization of macrophages, which are predominantly activated in the M2 subpopulation [60]. IPI-549, a selective PI3K-γ inhibitor, shifted GAMs from the M2 to the M1 phenotype by blocking PI3K-γ [61]. Using a TMZ-resistant glioma xenograft model, the combination of IPI-549 with PD-1 antibody strongly inhibited tumor growth, suggesting that macrophage repolarization could be a potential approach to overcome TMZ resistance [62].

Bevacizumab, a humanized monoclonal antibody to VEGF, is the only FDA-approved anti-angiogenic drug for GBM, which still meets resistance issues. Higher numbers of tumor-infiltrated GAMs are correlated with poor survival in antiangiogenic agent-treated patients, implying that GAMs participate in escape from antiangiogenic therapy [63]. Studies analyzed differential transcriptional expression between bevacizumab-resistant GBM patients and bevacizumab-naïve patients and suggested that macrophage migration inhibitory factor (MIF) was significantly downregulated and correlated with increased M2-like macrophages localizing to the tumor edge and tumor growth. Overexpression of MIF in bevacizumab-resistant GBM xenograft models resulted in decreased tumor weight, decreased GAMs, increased M1/M2 ratio, and decreased angiogenesis [64]. Above all, detailed mechanisms and underlying therapeutic strategies related to vascular–immune crosstalk need to be investigated further to realize synergetic effects of antiangiogenic therapy and immunotherapy in GBM.

Furthermore, several efforts have been made to elucidate the complex and interconnected GBM hallmarks. GAMs, as the most abundant immune cells in the TME, are largely responsible for the nature of the TME and play a critical role in the complicated and large network. The effects are achieved mainly through cytokines released by GAMs, such as VEGF, PDGF, bFGF, HGF, ILs, and MMPs. These multifunctional cytokines interact with each other and construct a protumoral TME. However, the underlying mechanisms for the role of GAMs in interconnected hallmarks of GBM still need further research [65].

## 6. GAM-Targeted Therapy in GBM

### 6.1. Targeting Phagocytosis Checkpoints

**Figure 3:**
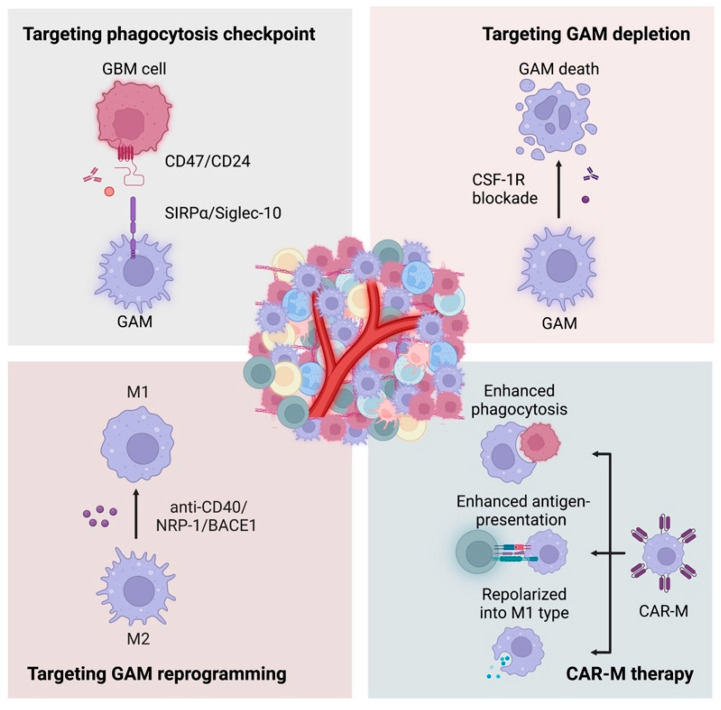
**GAM-targeted therapy in GBM.** Targeting the phagocytosis checkpoint is designed to block phagocytosis pairs, such as CD47/SIRPα and CD24/Siglec-10, to enhance the phagocytosis of tumor cells by macrophages; GAM depletion is aimed at reducing the number of GAMs in the TME. Targeting GAM reprogramming is to repolarize protumoral M2-like GAMs into tumor-suppressive M1-like macrophages; chimeric antigen receptor-macrophage (CAR-M) therapy is an approach using genetically engineered approaches to modify macrophages, which present enhanced phagocytosis, enhanced antigen presentation, and repolarization to M1-like macrophages.

Insufficient T-cell infiltration leads to unsatisfactory efficacy of immunotherapy checkpoint inhibitors (ICIs). Therefore, great attention has been given to macrophages, the most abundant immune cells in GBM, to reverse the immune “cold” environment (Figure 3).

A wide array of preclinical and clinical evidence has highlighted that phagocytosis checkpoints could be a potential target to induce an effective anticancer immune response of macrophages (Table 1). A series of phagocytosis checkpoint pairs have been identified, such as CD47/SIRPα, CD24/Siglec-10, PD-1/PD-L1, and MHC I-LILRB. Among them, CD47-SIRPα is the most well-studied phagocytosis checkpoint on macrophages to mediate “do not eat me” signaling [66,67]. During interactions between glioma and GAMs, CD47 has been found to be highly expressed in GBM cells [68,69]. The increased expression promotes proliferation and invasion of GBM cells and was positively correlated with glioma grade and negatively associated with clinical outcomes. CD47 is a transmembrane protein widely expressed on the surface of normal cells and solid tumors [70]. Structurally, it consists of an N-terminal extracellular variable region, five hydrophobic transmembrane helices, and a very short intracellular signal sequence. SIRPα is mainly expressed on the surface of myeloid cells (e.g., monocytes, macrophages, granulocytes), and its intracellular domain contains an immunoreceptor tyrosine inhibitory motif (ITIM). Binding of CD47 in normal cells and SIRPα on macrophages can cause phosphorylation of two SIRPα cytosolic ITIMs, which in turn recruit and activate Src homology-2 (SH2)-containing protein tyrosine phosphatases (SHP)-1/2 protein, leading to dephosphorylation of a series of intracellular proteins, inhibiting cytoskeleton rearrangement and cell motility, ultimately inhibiting macrophage phagocytosis and further impairing the antigen presentation and activation of adaptive immune responses [71,72]. Therefore, upregulated CD47-SIRPα signaling weakens phagocytosis of macrophages as an immune evasion mechanism. The blockade of CD47-SIRPa signaling significantly enhances phagocytosis by macrophages.

Therapeutic antibodies were invented, including Hu5F9-G4 [73]. Treating GBM with Hu5F9-G4 resulted in increased macrophage-mediated phagocytosis of GBM cells and GSCs and promoted the M1-phenotypic transition of macrophages [74]. In addition, Hu5F9-G4 significantly suppressed tumors and prolonged survival time in an immunocompetent allograft glioma mouse model [75,76]. In addition, the safety, pharmacokinetics, and pharmacodynamics of Hu5F9-G4 have been investigated in phase I clinical trials in adult patients with solid tumors (NCT02216409). The results showed that a treatment regimen for Hu5F9-G4 is well tolerated in patients with solid tumors and lymphoma. Another ongoing phase I clinical trial testing the safety of Hu5F9-G4 in patients with recurrent or progressive malignant brain tumors is currently recruiting participants (NCT05169944). Hu5F9-G4-mediated phagocytosis of GBM cells can even be significantly enhanced by irradiation and TMZ chemotherapy [77].

In addition to anti-CD47 antibodies, small molecule inhibitors targeting the CD47-SIRPα interaction or inhibiting CD47 expression are also being investigated. Compared with therapeutic antibodies, small molecule inhibitors have a low molecular weight [78] and enhanced permeability and retention effects [79,80,81], which might make them better candidates for brain cancer therapy. At present, the application of CD47 small molecule inhibitors in GBM remains under preclinical evaluation. RRx-001, a multipotent small molecule with vascular normalization effects, downregulated CD47 expression by inhibiting its transcription factor MYC directly [82,83]. A recent study demonstrated that administration of RRx-001 prior to TMZ or irinotecan results in significantly increased uptake of irinotecan and temozolomide in orthotopic glioma tumors of mice [84]. Other small molecule inhibitors, such as Pep-20, D4-2, and NCGC00138783, can directly block the binding of CD47 and SIRPα; [85,86,87] metformin, JQ1, and 4Mu can not only inhibit the CD47-SIRPα bond but also reduce CD47 expression at the transcriptional level [82,88,89,90]; PQ912 and SNE177 decrease CD47 expression by regulating posttranslational modification [91]; the widely used EGFR-TKI gefitinib, as a first-line treatment for patients with advanced EGFR mutation-positive non-small cell lung cancer, has also been shown to induce CD47 downregulation in vitro [92].

Similar to CD47, CD24 is overexpressed in a variety of solid tumors, including triple-negative breast cancer (TNBC), ovarian cancer, and GBM [93,94,95,96]. In glioma, CD24 expression levels are positively correlated with pathological grade and negatively correlated with outcomes [95]. Its partner Siglec-10 is highly expressed on tumor-associated macrophages with an ITIM in its cytoplasmic domain [97]. Some studies have confirmed that its expression is also increased in glioma and associated with poor prognosis of patients [98]. Mechanistically, the CD24-Siglec10 interaction induces the inhibition of phagocytosis in a similar way to CD47-SIRPa, which is mediated by SHP-1/SHP-2 once the ITIM region is phosphorylated [99].

Therapeutic strategies targeting other phagocytosis checkpoints are being explored. Amira A. Barkal et al. observed that both genetic ablation of CD24 or Siglec-10 and monoclonal antibody blockade of the CD24-Siglec-10 interaction robustly augmented the phagocytosis of all CD24-expressing human tumors tested. Dual treatment with CD24 and CD47 blocking antibodies revealed an increased induction of phagocytosis to nearly 30-fold that of baseline in some cancers [97]. Some anti-CD24 drugs targeting tumors have been put into clinical trials, such as SWA11 (mAb), which is related to ovarian cancer and pancreatic cancer, and rG7S-MICA (mAb), which is related to liver cancer. However, anticancer therapy using anti-CD24 antibodies in GBM to boost the innate immune system has not been tested in clinical trials. In summary, clinical and preclinical trials are mainly focused on targeting CD47/SIRPα and CD24/Siglec-10 to boost GAM phagocytic ability. More phagocytosis checkpoint pairs and related therapies are under development.

### 6.2. Targeting GAM Depletion

Colony-stimulating factor-1 receptor (CSF-1R) is a receptor tyrosine kinase expressed on macrophages that plays an important role in regulating the survival, proliferation, differentiation, and polarization of GAMs [100]. Binding of CSF-1R with its ligands, such as CSF-1 and IL-34, activates the CSF-1R pathway [101]. CSF-1R-positive macrophages correlate with poor prognosis in various solid cancers [102]. Targeting CSF-1R can effectively reduce the number of GAMs in the TME and promote GAM repolarization, thus promoting the activation of cytotoxic T-cells, inhibiting tumor growth, and preventing glioblastoma recurrence [103,104,105,106,107,108]. The small molecule inhibitor PLX-3397 was assessed in recurrent glioblastoma in a phase II clinical trial. However, no significant survival benefit was observed with PLX3397 monotherapy [109]. Therefore, considering the complexity and heterogeneity of the GBM TME, a macrophage-targeted strategy alone would not be enough to induce potent antitumor effects, and thus, more research focused on combination treatment is ongoing. Some preclinical evidence has demonstrated the feasibility and potential of the combination of anti-CSF-1R with other therapies. Administration of anti-CSF-1R and anti-PD1 to treat glioma in a mouse model indicated prolonged survival [110]. In addition, radiotherapy is also expected to function synergistically with immunotherapy in GBM, as ionizing radiation-induced DNA damage and cell death may be able to enhance the immunogenicity of tumor tissues and activate an immune response. Studies conducted by Akkari L and colleagues identified GAM gene expression signatures of different stages after radiotherapy in murine gliomas and found that targeting GAM populations using a colony-stimulating factor-1 receptor (CSF-1R) inhibitor combined with radiotherapy substantially enhanced survival in preclinical models [54]. In addition, a phase 1b/2 clinical trial (NCT02880371) evaluated ARRY-382, another CSF-1R inhibitor, plus pembrolizumab in patients with advanced solid tumors. Unfortunately, recently published results still failed to show a significant clinical benefit [111]. In summary, as mentioned above, the number of GAMs in the GBM TME is negatively correlated with prognosis in GBM patients, and therapeutics aimed at GAM depletion have been explored in GBM. The most well-studied target is CSF-1R. Although some favorable clinical and preclinical results have been obtained, studies regarding the efficacy, side effects, and combination therapy of CSF-1R blockade are still needed in the future.

### 6.3. Targeting GAM Reprogramming

In addition to CSF-1R, there are many other key molecules that regulate the survival and functions of GAMs in the TME, which have been demonstrated to be potential targets in macrophage-based therapies. For example, CD40, a costimulatory molecule, is expressed in most immune cells, including monocytes, macrophages, DCs, B cells, and nonimmune cells, such as endothelial cells, epithelial cells, and tumor cells [112]. The ligand CD40 L is expressed on the surfaces of activated T-cells and macrophages. Activated CD40/CD40 L pathways have broad immunostimulatory effects on APCs, B cells [112], and T-cells [113]. For macrophages, repolarization is induced to a tumor-suppressive type characterized by the production and release of proinflammatory cytokines, including IL-1β [114], TNFa [115], and IL-6 [116]. Therapeutically, agonistic CD40 antibodies (αCD40) are used in various solid tumors. In glioma, studies based on diverse experimental models and treatment regimens showed inconsistent results of their effectiveness [117,118]. More exploration of prime combinatorial regimens and the target population will be needed in the future. In addition, neuropilin-1 (NRP-1) is a coreceptor for class III semaphorins (SEMA3s) and members of the vascular endothelial growth factor (VEGF) family, and its b1 domain can interact and augment the VEGF-A and TGFβ pathways, thus promoting the protumorigenic M2 polarization of GAMs in the TME [119,120]. Miyauchi J. T. et al. found that the small molecule inhibitor EG00229, which inhibits its b1 domain, blocks the polarization of macrophages and increases the number of proinflammatory TAMs in the TME, resulting in an inhibitory effect on tumor growth [121]. Moreover, it has also been found that inhibition of β-amyloid precursor protein cleaving enzyme 1 (BACE1) with MK-8931 can effectively reprogram pTAM to sTAM and promote macrophage phagocytosis of glioma cells [122]. Moreover, some existing widely used drugs for nonmalignant diseases were also found to have the ability to regulate macrophage-mediated antitumor immune responses in glioma. The antibiotic minocycline inhibits microglial MMP expression and attenuates glioma invasion. The phase 1 clinical trial (NCT01580969) indicated that the combination of minocycline with radiation and bevacizumab was well-tolerated in patients with recurrent GBM [123,124]. Cyclosporine A, an immunosuppressant drug, has shown efficacy in attenuating glioma tumor growth and angiogenesis by inhibiting microglial infiltration in an experimental murine model [125]. Propentofylline, a drug with purported neuroprotective effects, has also been shown to reduce tumor growth in GBM by directly targeting microglia [126]. As mentioned above, reprogramming GAMs into antitumoral types is a wise approach to manipulate. Therapies targeting CD40, NRP-1, BACE1, and some existing widely used drugs for nonmalignant diseases are found to repolarize GAMs and show some promising clinical significance. However, elucidating the complicated regulatory mechanisms is a prerequisite for developing novel therapies.

### 6.4. Chimeric Antigen Receptor-Macrophage (CAR-M) Therapy

As great success for chimeric antigen T-cell therapy has been achieved in hematological malignancies, an increasing number of studies have attempted to copy this success in solid tumors. However, the results are far behind the expectations. Especially for GBM, some factors that compromise the efficacy of CAR-T therapy are nonnegligible. The existence of the BBB and lack of lymphatic networks restrict CAR-T cells from penetrating the GBM TME, and the immunosuppressive glioma TME decreases the viability of CAR-T cells and neutralizes their effects. Target-specific CAR-T cells have limited cytotoxicity in the heterogeneous GBM TME. To overcome the challenges of T-cell-based CAR therapy, researchers have focused their attention on macrophages. Therefore, strategies for the transduction of CARs into macrophages are under research, which is an approach using genetically engineered approaches to modify macrophages. Compared to CAR-T therapy, CAR-Ms have two major advantages. Unlike the poor infiltration of T-cells, macrophages exist abundantly in the GBM TME. Compared to the rapid development into exhaustion phenotypes of CAR-T cells after infiltrating the TME, the phenotypic plasticity of macrophages makes them changeable when faced with environmental stimuli.

Similar to CAR-T cells, CAR-Ms consist of an extracellular antigen-binding domain, hinge region, transmembrane domain, and intracellular domain. Structurally, the intracellular domain includes CD3ζ as used in CAR-T cells, the γ subunit of Fc receptor (FcRγ), and multiple epidermal growth factor-like domains protein 10 (Megf10), which contain immunoreceptor tyrosine-based activation motifs (ITAMs) with the ability to transduce phagocytic signals in macrophages. Similar to second- and third-generation CAR-T cells, an additional signaling domain is designed to enhance phagocytosis by macrophages. Studies have shown that the addition of a PI3K-recruiting domain significantly enhances phagocytosis by macrophages [127].

Studies conducted by Morrisey et al. in 2018 and Klichinsky in 2020 et al. show that CAR-Ms are able to phagocytize target antigen-expressing tumor cells, repolarize macrophages toward the antitumor M1 phenotype, promote T-cell recruitment, and stimulate the cells of the adaptive immune system. CAR-Ms against HER2 suggested a significant decrease in the metastatic tumor burden and longer overall survival in the mouse model. To our knowledge, there are no studies related to the use of CAR-Ms in GBM. However, there are still sufficient reasons to develop CAR-Ms strategies to treat GBM. Induced by the immunosuppressive cytokines secreted by GBM cells, most GAMs polarize into M2-like subtypes. CAR-Ms have been shown to convert the protumoral M2 phenotype into the proinflammatory M1 phenotype, which subsequently makes the GBM immune microenvironment “cold” into “hot”. GBM is an ideal candidate for this novel approach. Based on the normalized immune microenvironment, harnessing the immune system to defeat GBM will be easier to realize.

### 6.5. Other GAM-Based Therapies

Oncolytic virus (OV) is a novel way to elicit lytic tumor cell death to activate the immune response [128,129,130]. Beyond direct GBM cell lysis, the effect of OVs on macrophage modulation has been investigated. Van den Bossche, Wouter BL, and colleagues found that the oncolytic adenovirus Delta24-RGD, also known as DNX-2401, shifted the murine GBM macrophage phenotype from the pro-tumoral M2 toward the antitumoral and pro-inflammatory M1 phenotype, thereby disabling a major tumor-maintaining mechanism [131]. Recently, a phase I clinical trial of DNX-2401 treating patients with recurrent GBM suggested increased numbers of macrophages and proinflammatory factors, including IL-6 and TNF-α, in posttreatment tumor specimens [132]. Virus vector-mediated cancer gene therapy aimed at macrophage reprogramming is also under investigation. An adeno-associated virus (AAV) vector was used to selectively deliver antitumor transgenes encoding secreted antitumor proteins to tumor stromal cells, including macrophages, which could repolarize the target macrophage and promote a proinflammatory phenotype within the TME [133].

The SRC proto-oncogene nonreceptor tyrosine kinase (SRC) signaling pathway is constitutively activated in hypoxia in GBM. The increase in SRC activity causes VEGF, MMPs, and TGF-β upregulation. SRC inhibition could improve the GAM-orchestrated immunosuppressive TME. A series of SRC tyrosine kinase inhibitors (STKIs) have been developed, such as dasatinib, PP2, SI221, and bosutinib. However, despite encouraging preclinical results, most clinical trials in GBM have failed thus far [134,135].

The existence of the BBB prevents 98% of drugs from reaching the brain, which contributes to the limited effectiveness of chemotherapy. Novel biomaterials have been explored to address this issue. In a recent study, a pH-sensitive nanocomposite micelle composed of TfR-T12-PEG-PLGA and TATH7-PEG-PLGA was automatically assembled as a novel drug delivery system for sending chemotherapeutic paclitaxel (PTX) and immunomodulator Toll-7 receptor agonist R837, which successfully delivered PTX and R837 through the BBB and was rapidly ingested by tumor cells and tumor-associated immune cells in an acidic tumor microenvironment with the assistance of transferrin receptor (TfR) [136]. Macrophages infiltrated in tumors decreased significantly, and the immunosuppressive phenotype was relieved, characterized by increased TNF-α and decreased TGF-β. More effective and precise vectors should be developed for treatment, and the efficiency also needs to be demonstrated in clinical research.

## 7. Conclusions

Harnessing the host immune system to achieve potent antitumor responses with mild adverse reactions is the ultimate goal to pursue. Considering the immunosuppressive nature of the glioma microenvironment and the failure of T-cell-based therapies in glioma treatment, macrophages, as the most abundant immune cell in the glioma TME, might be an appropriate candidate to target. Overall, it is clear that crosstalk between GAMs and glioma plays a crucial role in glioma progression, angiogenesis, and treatment resistance. Understanding the underlying molecular mechanisms is necessary to develop macrophage-targeted therapies for glioma. More attention should be focused on the novel mechanisms and GAM-based clinical translational research in the future.

## Figures and Tables

**Table 1:** Clinical trials of macrophage-targeted therapies in glioblastoma.

Category		Therapeutic Approach	Conditions	Phases	Enrollment	Study Number	Status
Phagocytosis Checkpoints Blockade	CD47	Magrolimab	Brain Cancer	Phase 1	24	NCT05169944	Not yet recruiting
		RRx-001	Brain Tumor	Phase 1	24	NCT04525014	Recruiting
		RRx-001	Newly Diagnosed GBM	Phase 1	19	NCT02871843	Completed
		IBI188	Advanced Malignancies	Phase 1	49	NCT03717103	Completed
		AK117	Neoplasms Malignant	Phase 1	162	NCT04728334	Recruiting
		AK118	Neoplasms Malignant	Phase 1	159	NCT04349969	Not yet recruiting
		HX009	Advanced Solid Tumor	Phase 1	21	NCT04097769	Active, not recruiting
		IBC0966	Advanced Malignant Tumors	Phase 1	228	NCT04980690	Not yet recruiting
		IBI322	Advanced Solid Tumor	Phase 1	36	NCT04912466	Not yet recruiting
		IBI323	Advanced Solid Tumor	Phase 1	218	NCT04328831	Recruiting
		IBI324	Advanced Malignancies	Phase 1	45	NCT04338659	Not yet recruiting
		SRF231	Advanced Solid Cancers Hematologic Cancers	Phase 1	148	NCT03512340	Completed
		STI-6643	Solid Tumor Relapsed Solid Neoplasm Refractory Tumor	Phase 1	24	NCT04900519	Recruiting
		TQB2928	Advanced Cancer	Phase 1	180	NCT05192512	Recruiting
	SIRPα	BI 765063	Solid Tumor, Adult	Phase 1	116	NCT03990233	Recruiting
		CC-95251	Neoplasms	Phase 1	230	NCT03783403	Recruiting
GAM-depletion	CSF-1R	Cabiralizumab	Malignant Glioma, and other solid tumors	Phase 1	313	NCT02526017	Completed
		PLX3397	Recurrent GBM	Phase 2	38	NCT01349036	Terminated
		PLX3397	Newly Diagnosed GBM	Phase 1/2	65	NCT01790503	Completed
		ARRY-382	Advanced Solid Tumors	Phase 2	82	NCT02880371	Completed
	CXCR4	USL311	Relapsed/Recurrent GBM	Phase 2	26	NCT02765165	Terminated
Other GAM-based therapy	Oncolytic virus	DNX-2401	Recurrent High-Grade Glioma	Phase 1	36	NCT03896568	Recruiting
			Brainstem Glioma	Phase 1	24	NCT03178032	Active, not recruiting
			GBM	Phase 1	37	NCT02197169	Completed
			Recurrent Malignant Gliomas	Phase 1	37	NCT00805376	Completed
			Recurrent GBM	Phase 1	31	NCT01956734	Completed
			GBM, and other brain tumors	Phase 2	49	NCT02798406	Completed

## Data Availability

Data sharing is not applicable.
